# Improving Clinical Workflows: A Quality Improvement Project on Admission Documentation Standardization

**DOI:** 10.7759/cureus.94052

**Published:** 2025-10-07

**Authors:** Aarsh Sathia, Vikrant Ghadge

**Affiliations:** 1 General Surgery, Bharatratna Dr Babasaheb Ambedkar Municipal General Hospital, Mumbai, IND; 2 Internal Medicine, Surrey and Sussex Healthcare NHS Trust, Redhill, GBR

**Keywords:** admission documentation, pdsa, proforma, quality improvement, surgical admissions

## Abstract

Introduction

Complete admission notes are critical for patient safety and care continuity. A baseline audit of 40 surgical admission records at our tertiary hospital found major omissions: only 60% documented History of Presenting Illness (HOPI), 20% recorded Past Medical History (PMH), 60% captured Examination findings, and 47.5% included a Visual Aid. We implemented a standardized admission proforma to improve documentation completeness.

Methods

In April-May 2025, we conducted two Plan-Do-Study-Act (P.D.S.A.) cycles. Baseline data were collected by chart review of 40 consecutive admission notes. Cycle 1 introduced a first-version proforma; notes were reaudited (n = 40). Cycle 2 refined the form based on user feedback; a second reaudit was performed (n = 40). Key fields - HOPI, PMH, Examination, and Visual Aid - were scored as present or absent.

Results

After Cycle 1, HOPI rose to 100%, Examination to 97.5%, PMH to 30%, and Visual Aid fell to 27.5%. Following Cycle 2, HOPI and Examination reached 100%, PMH improved to 85% (*p*<0.001 vs baseline), and Visual Aid rose to 75% (*p*=0.022 vs baseline). Investigations and Management Plan were already at 100% throughout.

Conclusions

Iterative introduction of a structured admission proforma significantly enhanced documentation completeness. By Cycle 2, all targeted domains except Visual Aid met or exceeded 85% completeness. Standardized templates can reliably improve surgical admission notes and should be adopted broadly.

## Introduction

In our busy regional hospital’s General Surgery unit, new patient admissions were documented on blank doctor's orders sheet. Thorough documentation at admission is critical for safe, continuous patient care, but in practice, important details often get overlooked. Incomplete or fragmented documentation increases clinician workload and can directly threaten patient safety [1]. Incomplete admission notes frequently led to delay or postponement of surgical procedures. Prior studies suggest that structured admission templates can significantly improve documentation quality and completeness [2,3]. In our department we identified significant gaps in key admission fields (e.g. history of present illness, past medical history, physical examination findings). We therefore undertook a quality improvement (QI) initiative to implement revised, standardized admission templates using two Plan-Do-Study-Act (P.D.S.A.) cycles.

Background

Incomplete or inconsistent admission documentation poses significant risks to patient safety and continuity of care. Complete notes ensure effective communication among healthcare providers, thereby raising treatment standards and reducing errors [4]. In our surgical department, a baseline audit of 40 consecutive inpatient admissions revealed that key admission fields were frequently missing: only 60% of notes had a coherent History of Presenting Illness (HOPI), 20% documented Past Medical History, 60% included comprehensive Examination findings, and just 47.5% provided a Visual Aid (e.g., diagram/sketch) to support examination details. These gaps contribute to delays in medication reconciliation. Prior studies (e.g., Akasha et al. [5]; Tuffaha et al. [6]) indicate that standardized proformas and structured templates can markedly improve documentation completeness. There are reduced chances of medico-legal consequences in response to clinical errors if notes are accurate and complete [7]. Motivated by these findings and local safety concerns, our project aimed to implement and iteratively refine a standardized admission template to increase the proportion of fully documented admission notes.

Baseline measurements

Prior to intervention, a total of 40 surgical admission notes were recorded on free-form, unstructured paper sheets. House Officers wrote narratives HOPI and Past History without prompts, and physical Examination findings were noted in variable formats. Visual Aids were optional and infrequently used; there was no designated space for diagrams. Investigations and Initial Management fields were standardized and consistently completed. No formal training or template existed to guide residents on which fields to include. As a result, chart reviews showed wide variability: 40% of notes lacked a clear HOPI narrative, 80% omitted a detailed Past History, 40% lacked complete Examination findings, and over half (52.5%) contained no Visual Aid. Data collection at baseline used a simple audit form, classifying each field as Complete (“Y”), Incomplete (“I”), or Not Present (“N”).

Aims

Primary aim of this project is to standardize IPD admission notes in surgery department at a tertiary care setting. Secondary aim is to assess the outcome in terms of completeness of admission notes after implementation of standardized notes.

## Materials and methods

Setting and context

The study design is an Interventional P.D.S.A. Study was conducted in surgery ward of a tertiary care centre. Study duration is from 01 April, 2025 to 02 May, 2025. Baseline audit reviewed 40 consecutive inpatient admission notes.

We employed a two-cycle P.D.S.A. framework to design and refine a structured admission template.

P.D.S.A.-1

**Figure 1:**
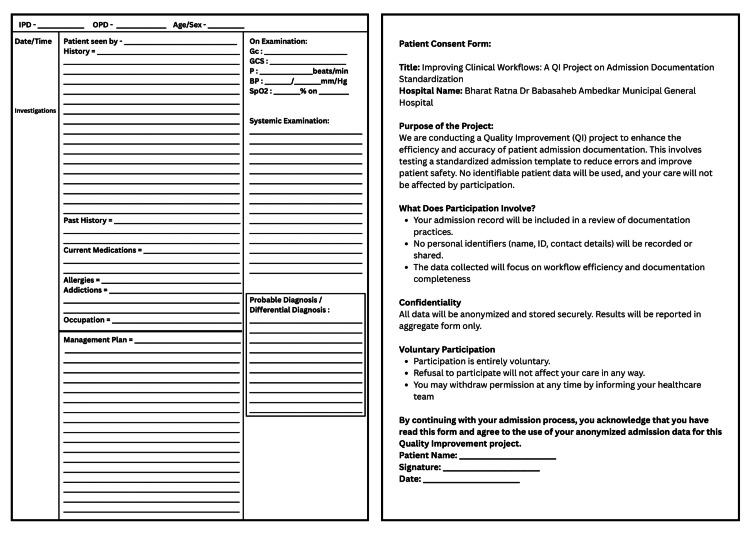
Admission template for first Plan-Do-Study-Act (P.D.S.A.) cycle.

Plan: Drafted a one-page paper proforma with prompts for each targeted field: IPD/OPD number, Age/Sex, Date/Time, HOPI, Past History, Current Medications, Allergies, Addictions, Occupations, Examination, Systemic Examination, Investigations, Probable Diagnosis and Management Plan. An informed consent was taken from the relatives and the patients. The template is shown in Figure 1.

Do: Introduced the Cycle-1 template during a 15-minute orientation session and printed 50 copies of the template. Residents and House Officers began using it immediately. Collected 40 consecutive admission notes over one week.

Study: Audited Cycle-1 notes with the same “Y/I/N” form: HOPI and Examination fields rose to 100% and 97.5% complete, respectively; Past History improved modestly to 30%; Visual Aid use unexpectedly dropped to 27.5%.

Act: The patient name section was manually struck through since no patient-identifiable data had to be collected. Feedback by Resident and House Officers was as follows: Font used on the form was very small. Not enough space to write comfortably and it led to increased time consumption to complete form and increased chances of error.

The following changes were finalized to be made in the second cycle: The font size was increased in the template for the second cycle and another section for Visual Aid was added to improve data collection.

P.D.S.A.-2

**Figure 2:**
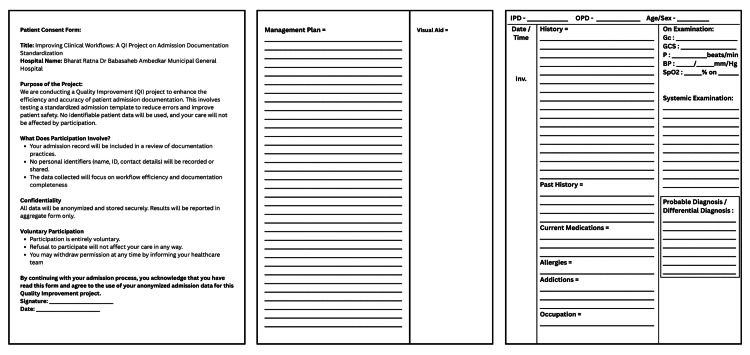
Admission template for second Plan-Do-Study-Act (P.D.S.A.) cycle.

Plan: Font was increased for the template of the second cycle. Another section for Visual Aid was added to improve data collection. The patient name section was removed from the consent page. The template is shown in Figure 2.

Do: Rolled out the Cycle-2 template and conducted a refresher orientation session during departmental meetings. Findings of first cycle were also discussed during the meeting. Printed 50 copies of the updated template. Collected 40 consecutive admission notes over one week.

Study: Audited Cycle-2 data: HOPI and Examination reached 100% each; Past History jumped to 85% complete; Visual Aid usage rebounded to 75%.

Act: Finalized the Cycle-2 template for adoption. We planned to discuss further P.D.S.A. cycles.

Analysis

For each documentation field, we calculated the proportion of records classified as complete. Comparisons between baseline and Cycle-2 were performed using chi-square tests. A p-value of <0.05 was considered statistically significant. Statistical analyses were performed using SPSS V.27 (Trial Version) Software (IBM Corp., Armonk, NY, USA).

Ethics

This QIP was reviewed by the Institutional Ethical Committee (IEC) and determined to be a local quality improvement activity; therefore, it was exempt from formal IEC approval. All patient data were de-identified prior to analysis. No external funding was received for this work.

Disclosures

No external funding was received for this work. The authors declare no competing interests.

## Results

Documentation completeness improved markedly for most fields after the interventions. HOPI documentation was recorded in 24/40 cases (60%) at baseline and reached 40/40 (100%) after the first cycle, which was sustained in Cycle-2. Examination findings were documented in 24/40 (60%) at baseline, improving to 97.5% (39/40) after Cycle-1 and 40/40 (100%) after Cycle-2. Past history documentation increased from only eight cases (20%) at baseline to 12/40 (30%) in Cycle-1 and 34/40 (85%) in Cycle-2. Use of visual aids (diagrams or annotated drawings) was 19/40 (48%) at baseline, dropped to 11/40 (28%) in Cycle-1, and then rose to 30/40 (75%) in Cycle-2. Notably, Investigations and Initial Management fields were documented in all records (100%) at every stage, reflecting already-standardized practice. 

After Cycle-1, the template substantially boosted completion of HOPI and examination fields, but did not initially improve visual aid usage. The second-cycle template (refined based on feedback) achieved further gains, especially in past history and visual aids, surpassing baseline levels. Overall, three of four targeted fields (HOPI, examination, past history) were documented in at least 85% of notes. These trends are summarized in Table 1 and Table 2.

**Table 1:** Documentation completeness across baseline and Plan-Do-Study-Act (P.D.S.A.) cycles.

Standard	Baseline 1st audit (%)	Cycle 1 reaudit (%)	Cycle 2 repeat reaudit (%)
History of Presenting Illness (HOPI)	60.0%	1	100.0%
Past Medical History	20.0%	30.0%	85.0%
Examination Findings	60.0%	97.5%	1
Visual Aid (Diagram)	47.5%	27.5%	75.0%
Investigations	1	1	1
Initial Management Plan	1	1	1

**Table 2:** Change in documentation completeness across Plan-Do-Study-Act (P.D.S.A.) cycles.

Field	Baseline % (n=40)	Cycle 1 % (n=40)	Cycle 2 % (n=40)	Absolute Δ (Base→C2)	p-value (Base vs C2)
History of Presenting Illness (HOPI)	24 (60.0%)	40 (100.0%)	40 (100.0%)	40.0 pp	<0.001
Past Medical History	8 (20.0%)	12 (30.0%)	34 (85.0%)	65.0 pp	<0.001
Examination Findings	24 (60.0%)	39 (97.5%)	40 (100.0%)	40.0 pp	<0.001
Visual Aid (Diagram)	19 (47.5%)	11 (27.5%)	30 (75.0%)	27.5 pp	0.022
Composite Score (mean ± SD)	1.63 ± 1.17	2.40 ± 0.94	3.00 ± 0.77	+1.37	

## Discussion

The introduction of structured admission templates was associated with a clear improvement in documentation completeness. At baseline, key elements such as examination findings and initial management plans were often omitted. Implementing the first P.D.S.A. cycle (a structured clerking form) produced large gains: most documentation domains showed higher completion rates than at baseline. Importantly, Cycle-2 (a refined template) either maintained or further improved these gains, suggesting that the improvement was not only rapid but also sustainable. In essence, each cycle’s audit showed progressively more complete admission notes, reflecting effective incorporation of structured templates into clinical practice.

These findings align with prior literature on structured admissions documentation. For example, Ehsanullah et al. [8] found that a surgical admissions proforma significantly increased documentation across many fields. In our work, we observed similar gains in past history, medication lists, vital signs, and patient instructions after introducing templates. Likewise, an audit by Umar et al. [9] reported that a surgical clerking proforma improved 28 of 33 documentation criteria and was strongly preferred by clinical staff. These parallels reinforce that structured admission forms reliably enhance the quality and completeness of clinical notes. The high uptake and positive feedback in our project mirror these reports, underscoring that well-designed templates are accepted and valued by healthcare teams.

The gradual improvements across cycles suggest a durable change in clinician behavior. Our data indicate that once the proforma was integrated into workflow, better documentation persisted and even advanced. This sustainability likely reflects both the embedding of the template in practice and the usability improvements from the second cycle. Such lasting effects have broader implications: standardized admission forms can continuously prompt thorough assessments and can be adapted in other units or hospitals. As Umar et al. [9] noted, “this simple intervention could be replicated to improve the admissions process” in diverse settings. In the end, more complete admission notes should improve handovers, facilitate audits, and contribute to patient safety and efficiency.

## Conclusions

This QI project demonstrated that introducing and iterating standardized admission templates significantly improved documentation completeness. After two P.D.S.A. cycles, all admission records had complete HOPI and examination sections, and past medical history documentation rose to 85%. The decline then rise in visual-aid documentation highlights the importance of iterative design and staff feedback. These findings align with prior studies showing that structured proformas enhance documentation.

Limitations include a small sample size and single-center design, which may limit generalizability. We recommend continued use of the revised templates, regular audit of admission notes, and staff education to sustain gains. Future cycles could focus on fields that lagged behind and explore electronic form prompts. Embedding these standardized templates into routine practice would help maintain high-quality admission documentation and ultimately support better patient care.
